# Eliminating Ambiguous Treatment Effects Using Estimands

**DOI:** 10.1093/aje/kwad036

**Published:** 2023-02-14

**Authors:** Brennan C Kahan, Suzie Cro, Fan Li, Michael O Harhay

**Keywords:** estimands, estimates, estimators, randomized trials, treatment effects

## Abstract

Most reported treatment effects in medical research studies are ambiguously defined, which can lead to misinterpretation of study results. This is because most authors do not attempt to describe what the treatment effect represents, and instead require readers to deduce this based on the reported statistical methods. However, this approach is challenging, because many methods provide counterintuitive results. For example, some methods include data from all patients, yet the resulting treatment effect applies only to a subset of patients, whereas other methods will exclude certain patients while results will apply to everyone. Additionally, some analyses provide estimates pertaining to hypothetical settings in which patients never die or discontinue treatment. Herein we introduce *estimands* as a solution to the aforementioned problem. An estimand is a clear description of what the treatment effect represents, thus saving readers the necessity of trying to infer this from study methods and potentially getting it wrong. We provide examples of how estimands can remove ambiguity from reported treatment effects and describe their current use in practice. The crux of our argument is that readers should not have to infer what investigators are estimating; they should be told explicitly.

## Abbreviations

ICH-E9(R1): International Council for Harmonisation of Technical Requirements for Pharmaceuticals for Human Use Efficacy Guideline 9, Revision 1

Results from randomized trials and observational studies are used to inform policy decisions about which interventions to use. However, reported treatment effects in these studies are often ambiguously defined, which can lead to misinterpretation of study results (1, 2). This is because most studies do not attempt to describe what the treatment effect represents, and instead require readers to deduce this based on the reported statistical methods (1, 2). However, this approach is challenging, because many methods provide counterintuitive results (Table 1).

**Table 1:** Standard Statistical Estimators Which Can Lead to Unexpected Estimands

**Estimator**	**Estimand Being Estimated**
Cox model for a nonfatal outcome (e.g., hospital admission), where participants who die are censored at the point of death (where death is the intercurrent event)	*Hypothetical strategy* (i.e., the hazard ratio in the hypothetical setting where participants do not die).
	The reason the Cox model estimates a hypothetical strategy in this setting is that it makes the implicit assumption that censored participants are still alive and at risk of the outcome (hospital admission). This model assumes that 1) the hypothetical setting is well-defined (i.e., we can describe how participants would no longer die) and 2) there is no unmeasured confounding between the occurrence of the intercurrent event and outcomes.
Mixed models for repeated measures, where outcome data are not collected after participants die (where death is the intercurrent event)	*Hypothetical strategy* (i.e., the treatment effect in the hypothetical setting where participants do not die).
	The reason for this is that outcome data will be missing after death (as they do not exist, and so cannot be measured), and the mixed model for repeated measures implicitly imputes what the missing data would have been had participants been alive. This model assumes that 1) the hypothetical setting is well-defined (i.e., we can describe how participants would no longer die) and 2) there is no unmeasured confounding between the occurrence of the intercurrent event and outcomes.
Inverse probability weighting (where outcome data post–intercurrent event are excluded and the remaining data are weighted according to the inverse probability of not experiencing the intercurrent event)	*Hypothetical strategy* (i.e., the treatment effect in the hypothetical setting where participants did not experience the intercurrent event).
	The reason for this is that data collected after the intercurrent event occurs are implicitly replaced using data from participants who did not experience the intercurrent event. This model assumes that 1) the hypothetical setting is well-defined (i.e., we can describe how participants would no longer die) and 2) there is no unmeasured confounding between the occurrence of the intercurrent event and outcomes.
CACE analysis using all randomized patients	*Principal stratum strategy* (i.e., the treatment effect in the subpopulation of participants who would always adhere to treatment regardless of the assignment).
	The reason for this is that although CACE analysis is performed using the entire trial population, the results only apply to the subset of participants who would have adhered to treatment. Common CACE estimators, such as those based on instrumental variables, assume the absence of “defiers”—that is, participants who would receive the intervention if they were assigned to usual care but would not receive the intervention if assigned to the active intervention group.
Modified intention-to-treat analysis where participants who do not begin treatment are excluded	*Principal stratum strategy* ^a^ (i.e., the treatment effect in the subpopulation of participants who would begin either treatment).
	By excluding participants who do not begin treatment, this approach estimates the effect in those who *do* begin treatment. This estimator assumes that the intercurrent event (treatment initiation) is not affected by randomized treatment arm (i.e., patients who begin treatment in one treatment group would also do so in the other group, and vice versa) (8).

Abbreviation: CACE, complier average causal effects.

^a^ This analysis can also estimate a *hypothetical* strategy under an alternative (stronger) set of assumptions.

For example, in a recent trial, Wollenberg et al. (3) reported that baricitinib significantly reduced impairment in daily activities for patients with atopic dermatitis. All randomized participants were included in the analysis in their randomized group, so readers might expect the treatment effect to correspond to the intervention’s effect if introduced as part of routine practice. However, the reported effect actually corresponded to a hypothetical setting in which participants who stopped treatment had instead continued and rescue therapy was denied (1). This interpretation is due to the statistical methods employed, where outcome data recorded after patients used rescue therapy or stopped taking treatment were set to missing, and then a mixed model for repeated measures was used to implicitly impute what outcomes would have been had participants not discontinued treatment or received rescue therapy.

Regardless of one’s preference for a treatment effect corresponding to usual practice versus one corresponding to a hypothetical situation, it is clear that the treatment effect’s interpretation should be reported so that readers can understand the exact question being addressed in the trial. However, the interpretation for the baricitinib trial was not given in the published article (3), and instead readers were left to sift through and interpret complex statistical methods to understand what exactly was being estimated.

## AMBIGUITY IN REPORTED TREATMENT EFFECTS

The problems described above are not unique (1, 2). In a recent review of randomized trials published in 2020, Cro et al. (1) found that 98% of trial reports did not attempt to describe what the reported treatment effect represented. Instead, readers were left to deduce these interpretations based on the reported study methods.

However, this approach is challenging. In 54% of the trials analyzed by Cro et al., it was impossible to deduce what exactly was being estimated from the reported methods (1). There are several reasons for this. First, statistical methods are often insufficiently described (4–6). In approximately 15% of trial reports, it is impossible to ascertain exactly what statistical analyses were performed (4).

Second, the statistical methods (even when reported in precise detail) are not always sufficient to uniquely identify which treatment effect is being estimated (2, 7). For instance, consider a trial in which the analysis population is restricted to persons who complied with their allocated treatment (often referred to as a per-protocol analysis). Does this analysis estimate the treatment effect that would have been observed in the entire trial population had all patients complied with their allocated treatment? Or does it estimate the treatment effect in the subset of trial participants who would have complied with their allocated treatment? In fact, investigators may use this analysis approach to estimate either treatment effect, depending on which assumptions they make, so readers are unable to deduce what investigators have intended unless they are told explicitly (8).

Finally, it is often difficult to understand what is being estimated from advanced statistical procedures, unless readers are proficient in the particular method used. This is particularly the case when certain aspects of the estimator do not match those of the estimand—for instance, when statistical methods use data from an analysis population that is not the same as the population for which the treatment effect applies. For example, complier average causal effects (CACE) analysis (9) uses data from the full trial population, but the resulting treatment effect only applies to the subpopulation of participants who would have complied with the intervention (9). Conversely, some statistical methods exclude participants who did not comply with treatment, yet estimate the treatment effect in the entire study population, under hypothetical compliance (10).

## UNRAVELING AMBIGUOUS TREATMENT EFFECTS

In 14% of trials published in 2020 that were included in the recent systematic review (1) and 74% of those submitted for regulatory approval between 1996 and 2017, hypothetical “what if?” treatment effects were used (11). Hypothetical “what if?” treatment effects describe what would have happened in hypothetical settings—for instance, if patients who forgot to take their daily medication had instead remembered to. However, in only 2 of the published trials did authors explain that their reported treatment effect related to a hypothetical scenario. For the other trials, correct interpretation of results required readers to infer from study methods that the treatment effects pertained to a hypothetical scenario, provided they had sufficient expertise to do so (1).

“What if?” treatment effects can be useful for informing clinical practice. For instance, patients who forget to take medication can be sent a reminder; or sometimes the reason treatment was discontinued will not apply in the future (e.g., treatment interruptions due to the coronavirus disease 2019 (COVID-19) outbreak (12–14)). However, many trials use “what if?” situations where the clinical relevance is less clear. For instance, in the recent review (1), 24% of “what if?” scenarios related to the setting where patients who were forced to stop taking treatment due to adverse effects instead continued to take medication despite the adverse effects; and in 79% of trials where deaths occurred but were not considered part of the outcome measure, investigators estimated what the treatment effect would have been in the hypothetical setting where those patients who died had lived instead.

None of these trials gave any indication of how patients might continue taking the assigned treatment despite the adverse effects, or how mortality might be completely eliminated in their “what if?” scenario (1). For instance, would patients be forced to continue taking treatment despite toxicity, or were investigators imagining a setting where the treatment had somehow become less toxic? These scenarios address fundamentally different questions, and the validity of each may depend on the plausibility of the assumptions about *how* the hypothetical setting envisaged may occur (15).

## LIMITATIONS OF CURRENT REPORTING GUIDELINES

Readers need to understand *what* is being estimated to make informed decisions about the evidence. Yet, this information is rarely reported in published articles or protocols (1, 2). This may be partly due to current reporting guidelines, which do not mandate the provision of this information (16–19). Instead, reporting guidelines typically require precise descriptions of the study methods, such as the eligibility criteria, analysis populations, treatment arms, and statistical methods; however, as we described above, telling us *what* was done does not necessarily tell us what the treatment effect *means*. For instance, providing information about the study’s eligible population or analysis population does not necessarily tell us the population the treatment effect corresponds to, and similar issues exist with trying to identify the treatments being compared or even the outcome measure being used in the estimation of the treatment effect (Table 2).

**Table 2:** Core Elements of an Estimand

**Attribute of Estimand**	**Description**	**Limitations of Current Reporting Requirements**
Population	The population of patients we want to estimate the treatment effect for	Reporting guidelines require authors to report the patient eligibility criteria and the analysis population; however, neither of these tell us *who* the treatment effect applies to. For instance, certain statistical methods use data from all randomized patients but estimate the treatment effect for a subpopulation of patients. Other methods that exclude certain patients (e.g., those with treatment deviations) estimate a treatment effect that applies to *all* participants under hypothetical compliance.
Treatment conditions	The treatment conditions being compared for the treatment effect	Although most reporting guidelines require a description of the treatments patients are assigned to, these are not necessarily the same as the versions of treatment being compared for the treatment effect. For example, some protocols may allow patients to discontinue treatment or receive rescue medication, but use statistical methods which estimate the treatment effect for a version of treatment where patients did not discontinue treatment or receive rescue medication.
Endpoint	The outcome measure collected for each patient that the treatment effect is based on	The outcome measure on which data are collected for each patient is a key component of all reporting guidelines; however, this is not always the outcome measure used for the treatment effect. This mismatch can occur when there are intercurrent events (see row below), particularly death (if death is not inherently part of the outcome measure). For example, for the outcome “disease recurrence up to 12 weeks,” it is not entirely clear what outcome should be used in the treatment effect if a patient dies in week 7 without recurrence. The outcome used in the treatment effect could be either “recurrence up to 12 weeks or death, whichever is sooner” or “recurrence or death up to 12 weeks” (both of which would lead to a different outcome for this patient).
Summary measure	The summary measure used to compare endpoints between treatment conditions for the treatment effect (e.g., mean difference, risk ratio, odds ratio, etc.)	The summary measure can typically be inferred from the statistical model used (e.g., a difference in mean values from a linear regression model or an odds ratio from a logistic regression model), though this is not always the case. Sometimes advanced statistical models are used to compute risk ratios or risk differences from logistic models (whose regression parameters correspond to odds ratios).
Handling of intercurrent events^a^	How postbaseline events, which affect the interpretation or occurrence of the endpoint (e.g., treatment discontinuation, treatment switching, use of rescue medication, or death if not defined as part of the outcome), are handled in the definition of the treatment effect	Reporting guidelines do not typically require handling of intercurrent events to be reported; however, the handling of such events is critically important to interpretation of the treatment effect. For instance, how we handle “treatment discontinuation” can lead to drastically different treatment effects, ranging from “the effect regardless of discontinuation” to “the effect in the subset of patients who would not discontinue” or “the effect in the hypothetical setting where patients would not discontinue.”

^a^ Standard strategies for handling intercurrent events are 1) treatment policy (we are interested in the treatment effect regardless of the intercurrent event); 2) hypothetical (a hypothetical scenario is envisaged where the intercurrent event does not (or does) occur; we are interested in the treatment effect if participants did not (or did) experience the intercurrent event); 3) composite (the intercurrent event is used as part of the endpoint definition); 4) principal stratum (we are interested in the treatment effect in the subpopulation of patients who would (or would not) experience the intercurrent event); and 5) while-on-treatment (the endpoint measured up to the occurrence of the intercurrent event is of interest; this is referred to as “while alive” when the intercurrent event is death).

The crux of the issue comes down to this: Readers should not have to infer what investigators are estimating; they should be told explicitly (20).

## ESTIMANDS AS A SOLUTION

*Estimands* present a solution to the aforementioned issue (2, 7, 11, 15, 21–25). An estimand is a clear description of what the treatment effect represents, thus saving readers the necessity of trying to infer this from study methods (and potentially getting it wrong). Estimands describe what happens to the same set of patients under different treatment conditions (i.e., they define a causal comparison between treatments based on a common target population (26)).

The concept of an estimand (the target of estimation) is not inherently new, but its use in randomized trials was only recently formalized with the publication of the International Council for Harmonisation of Technical Requirements for Pharmaceuticals for Human Use Efficacy Guideline 9, Revision 1 (ICH-E9(R1)) addendum in November 2019 (15). Estimands can be defined using a structured approach and are typically comprised of 5 attributes (Table 2): 1) the population of patients the treatment effect applies to, 2) the treatment conditions being compared, 3) the endpoint, 4) a summary measure of how endpoints will be compared between treatment conditions (e.g., through a risk ratio or risk difference), and 5) how intercurrent events (postbaseline events which affect interpretation or occurrence of outcomes, such as treatment discontinuation or treatment switching) are handled in the treatment effect definition (Table 3). In some settings, additional attributes may also be required (27–30).

**Table 3:** Different Strategies for Handling Intercurrent Events in the Definition of an Estimand^a^

**Strategy for Handling Intercurrent Events in Estimand Definition**	**Description**	**Definition Using Potential Outcomes**
Treatment policy	The outcome regardless of the occurrence of the intercurrent event is of interest (i.e., the intercurrent event is considered part of the treatment strategy). Cannot be used for truncating events which preclude the occurrence of the outcome.	$E\left(I\left\{{T}_Y^{\left(Z=1\right)}\le F\right\}-I\left\{{T}_Y^{\left(Z=0\right)}\le F\right\}\right)$
Composite	The intercurrent event is included in the endpoint definition—for example, by assigning a particular value of the endpoint to patients who experience the intercurrent event. Different composite estimands could be defined on the basis of the particular value assigned to the endpoint.	$E\left(I\left\{\min \left({T}_Y^{\left(Z=1\right)},{T}_R^{\left(Z=1\right)}\right)\le F\right\}-I\left\{\min \left({T}_Y^{\left(Z=0\right)},{T}_R^{\left(Z=0\right)}\right)\le F\right\}\right)$ ^b^
While on treatment/while alive	The endpoint prior to the occurrence of the intercurrent event is of interest.	$E\left(I\left\{{T}_Y^{\left(Z=1\right)}\le \min \left(F,{T_R}^{\left(Z=1\right)}\right)\right\}-I\left\{{T}_Y^{\left(Z=0\right)}\le \min \left(F,{T_R}^{\left(Z=0\right)}\right)\right\}\right)$
Hypothetical	The effect of treatment in a hypothetical scenario where the intercurrent event would not occur is of interest. There can be multiple hypothetical settings that would apply to any particular intercurrent event, so it is necessary to describe the precise hypothetical setting envisaged.	$E\left(I\left\{{T}_Y^{\left(Z=1,{R}^{\left(Z=1\right)}=0\right)}\le F\right\}-I\left\{{T}_Y^{\left(Z=0,{R}^{\left(Z=0\right)}=0\right)}\le F\right\}\right)$ ^b,c^
Principal stratum	The treatment effect in the subset of participants who would (or would not) experience the intercurrent event is of interest. Different principal strata could be defined (e.g., the set of participants who would not discontinue treatment under either treatment vs. the set who would not discontinue if assigned to the intervention).	$E\left(I\left\{{T}_Y^{\left(Z=1\right)}\le F\right\}-I\left\{{T}_Y^{\left(Z=0\right)}\le F\right\}|{R}^{\left(Z=1\right)}={R}^{\left(Z=0\right)}=0\right)$ ^b^

^a^ $Z$ denotes treatment allocation (0 = control, 1 = intervention) and $Y$ denotes a binary outcome, defined as 1 if an event occurred within a defined follow-up period $F$ and 0 if an event did not occur by time $F$. Equivalently, $Y$ can be written as $I\left\{{T}_Y^{\left(Z=z\right)}\le F\right\}$, where ${T}_Y$ denotes the time at which the outcome $Y$ occurred, ${T}_Y^{\left(Z=0\right)}$ and ${T}_Y^{\left(Z=1\right)}$ denote the potential outcomes for the time of $Y$ under intervention and control conditions, respectively, and $I\left\{\cdot \right\}$ is an indicator variable (defined as 1 if the expression inside the brackets occurred and 0 otherwise). $R$ denotes an intercurrent event (1 = occurred, 0 = did not occur), with ${R}^{\left(Z=1\right)}$ and ${R}^{\left(Z=0\right)}$ representing the potential occurrences of the intercurrent event under intervention and control conditions and ${T}_R^{\left(Z=1\right)}$ and ${T}_R^{\left(Z=0\right)}$ denoting the potential times of the intercurrent event under intervention and control conditions.

^b^ For composite, hypothetical, and principal-stratum strategies, different versions of the estimand could be defined than those considered here (e.g., based on different values assigned to the outcome, different hypothetical settings, or different principal-stratum populations). For instance, an alternative principal-stratum estimand, where the population is defined as participants who would adhere to the intervention, is $E\left(I\left\{{T}_Y^{\left(Z=1\right)}\le F\right\}-I\left\{{T}_Y^{\left(Z=0\right)}\le F\right\}|{R}^{\left(Z=1\right)}=0\right)$.

^c^ In order for the hypothetical estimand to be well-defined, the specific setting under which the intercurrent event would not occur (i.e., under which $R=0$) needs to be precisely defined concurrently, since the potential outcomes may differ depending on the exact setting imagined where $R=0$. This implies that impossible hypothetical scenarios, for which there is no possible setting in which $R=0$ for all patients, are generally not well-defined.

An example of how estimands remove ambiguity from treatment effects is provided in Table 4. In the atopic dermatitis trial introduced above (3), inferring the treatment effect’s interpretation based on the reported methods requires knowledge of how mixed-effects models can implicitly impute missing outcome data under hypothetical “what if?” scenarios. Conversely, the fact that the treatment effect pertains to a hypothetical scenario is explicitly described in the estimand, ensuring that readers understand the treatment effect even in the absence of detailed statistical knowledge. Examples of how estimands can be reported are shown in Web Appendices 1 and 2 (available at https://doi.org/10.1093/aje/kwad036) and Web Tables 1–3 (31).

**Table 4:** Understanding What Treatment Effects Represent Based on Reported Statistical Methods Versus Estimands in a Trial of Baricitinib for Atopic Dermatitis^a^

**Statistical Methods**	**Estimand**
“The analysis population comprised all randomised patients, regardless of whether they received the correct treatment” (3, p. 1545). “Mean change from baseline for continuous measures (PROMIS^b^ and WPAI-AD^c^) was evaluated using a restricted maximum likelihood-based mixed model repeated measures (MMRM), where the model includes treatment, region, baseline disease severity [validated Investigator Global Assessment for AD (vIGA-AD)^d^], visit and treatment-by-visit-interactions as fixed categorical effects and baseline and baseline-by-visit-interaction as fixed continuous effects” (3, p. 1546).	The treatment effect is the difference between mean values in the WPAI:AD change from baseline score (at 16 weeks) for baricitinib 4 mg or 2 mg versus placebo daily, plus topical corticosteroids for adults with atopic dermatitis (meeting the trial eligibility criteria), regardless of whether participants received the correct treatment, in the hypothetical scenario where treatment discontinuation did not occur regardless of side effects or other adverse effects and rescue therapy was not provided, even if medically indicated.
“Data collected after first rescue therapy or permanent study drug discontinuation were considered missing… No explicit imputations were conducted for continuous measures; MMRM analysis was performed to mitigate the impact of missing data because it yields valid inferences assuming that missing observations are missing-at-random” (3, p. 1546).	

Abbreviations: AD, atopic dermatitis; MMRM, mixed-model repeated measures; PROMIS, Patient-Reported Outcomes Measurement Information System; vIGA-AD, Validated Investigator Global Assessment Scale for Atopic Dermatitis; WPAI:AD, Work Productivity and Activity Impairment Questionnaire: Atopic Dermatitis.

^a^ Based on the study by Wollenberg et al. (3).

^b^ PROMIS Health Organization, River Forest, Illinois.

^c^ Created by Reilly et al. (41).

^d^ Eli Lily and Company, Indianapolis, Indiana.

At its heart, an estimand provides readers with a clear sense of what reported treatment effects represent.

## USE OF ESTIMANDS IN PRACTICE

Although the concept of estimands is not new, estimands have only recently started to gain widespread attention. Since the publication of the estimand framework in the ICH-E9(R1) addendum (15), medicine regulators in Europe, the United States, Canada, Singapore, China, Switzerland, and Taiwan have changed their policies to require companies submitting applications to include estimands, and currently regulators in Brazil, South Korea, and Japan are in the process of implementing this policy (32).

Notably, most reporting guidelines were written before the release of the ICH-E9(R1) addendum, which may partly explain why estimands are not featured. However, at least 1 recent reporting guideline has included specification of the target estimand as a required item (33). Furthermore, some published trial reports and protocols have begun using estimands to clarify reported treatment effects (34–37).

## USE OF ESTIMANDS IN NONEXPERIMENTAL RESEARCH

Because the primary goal of estimands is to increase the clarity of what reported treatment effects represent, they are just as important for observational studies as for randomized trials. All of the same principles discussed thus far still apply; however, there may be additional considerations for observational research (30).

For example, when using propensity score balancing weights to address measured confounding, different methods of doing so can target different populations, thus changing the “population” attribute of the estimand (38, 39). Specifically, the average treatment effect in the observed population is estimated via inverse-probability-of-treatment weights; the average treatment effect among the treated is estimated by multiplying the inverse-probability-of-treatment weights by the propensity score; and the average treatment effect in the overlap population (a population emphasizing clinical equipoise with the highest uncertainty in receiving both treatments) is estimated via the overlap weights (40). Thus, a clear description of not only what weighting scheme has been used but also what population it has targeted is essential to proper interpretation of study results.

Furthermore, different types of intercurrent events may occur in observational studies as compared with randomized trials. For instance, the protocol of a randomized trial may mandate that participants reduce their treatment dose in a specific manner when adverse events occur, while in observational studies, where treatment decisions are left to the treating physicians, some participants may stay on the same dose while others may stop treatment entirely or switch to an alternative. Additionally, many data sources that routinely collect data, such as registries, may not collect information on which participants experience certain intercurrent events. Thus, investigators in studies using these data sources may not be able to choose which strategy to apply for these events.

## WHAT SHOULD CHANGE GOING FORWARD

Despite the increased focus on estimands, their use in practice is still rare (1, 2). We believe the inclusion of estimands as a mandatory reporting item in reporting guidelines (including each of the 5 aspects encompassing the estimand) would have an enormous positive impact on increasing clarity around the interpretation of reported treatment effects in research studies.

In conclusion, estimands offer a simple way of clarifying the interpretation of reported treatment effects so as to avoid misinterpretations of study results.

## Supplementary Material

Web_Material_kwad036

## References

[1] Cro S , Kahan BC, Rehal S, et al. Evaluating how clear the questions being investigated in randomised trials are: systematic review of estimands. *BMJ*. 2022;378:e070146. 35998928 10.1136/bmj-2022-070146 PMC9396446

[2] Kahan BC , Morris TP, White IR, et al. Estimands in published protocols of randomised trials: urgent improvement needed. *Trials*. 2021;22(1):686. 34627347 10.1186/s13063-021-05644-4 PMC8500821

[3] Wollenberg A , Nakahara T, Maari C, et al. Impact of baricitinib in combination with topical steroids on atopic dermatitis symptoms, quality of life and functioning in adult patients with moderate-to-severe atopic dermatitis from the BREEZE-AD7 Phase 3 randomized trial. *J Eur Acad Dermatol Venereol*. 2021;35(7):1543–1552. 33834521 10.1111/jdv.17278 PMC8251919

[4] Cro S , Forbes G, Johnson NA, et al. Evidence of unexplained discrepancies between planned and conducted statistical analyses: a review of randomised trials. *BMC Med*. 2020;18(1):137. 32466758 10.1186/s12916-020-01590-1 PMC7257229

[5] Greenberg L , Jairath V, Pearse R, et al. Pre-specification of statistical analysis approaches in published clinical trial protocols was inadequate. *J Clin Epidemiol*. 2018;101:53–60. 29864539 10.1016/j.jclinepi.2018.05.023

[6] Kahan BC , Ahmad T, Forbes G, et al. Public availability and adherence to prespecified statistical analysis approaches was low in published randomized trials. *J Clin Epidemiol*. 2020;128:29–34. 32730852 10.1016/j.jclinepi.2020.07.015

[7] Mitroiu M , Teerenstra S, Oude Rengerink K, et al. Estimation of treatment effects in short-term depression studies. An evaluation based on the ICH E9(R1) estimands framework. *Pharm Stat*. 2022;21(5):1037–1057. 35678545 10.1002/pst.2214 PMC9543408

[8] Kahan BC , White IR, Edwards M, et al. Using modified intention-to-treat as a principal stratum estimator for failure to initiate treatment [published online ahead of print March 14, 2023]. Clin Trials. 2022. (10.1177/17407745231160074). PMC10262320 36916466

[9] Angrist JD , Imbens GW, Rubin DB. Identification of causal effects using instrumental variables. *J Am Stat Assoc*. 1996;91(434):444–455.

[10] Hernán MA , Robins JM. Causal Inference: What If. Boca Raton, FL: Chapman & Hall/CRC Press; 2020.

[11] Mitroiu M , Oude Rengerink K, Teerenstra S, et al. A narrative review of estimands in drug development and regulatory evaluation: old wine in new barrels? *Trials*. 2020;21(1):671. 32703247 10.1186/s13063-020-04546-1 PMC7376663

[12] Cro S , Morris TP, Kahan BC, et al. A four-step strategy for handling missing outcome data in randomised trials affected by a pandemic. *BMC Med Res Methodol*. 2020;20(1):208. 32787782 10.1186/s12874-020-01089-6 PMC7422467

[13] Kahan BC , Morris TP, White IR, et al. Treatment estimands in clinical trials of patients hospitalised for COVID-19: ensuring trials ask the right questions. *BMC Med*. 2020;18(1):286. 32900372 10.1186/s12916-020-01737-0 PMC7478913

[14] Van Lancker K , Tarima S, Bartlett J, et al. Estimands and their estimators for clinical trials impacted by the COVID-19 pandemic: a report from the NISS Ingram Olkin Forum Series on unplanned clinical trial disruptions. *Stat Biopharm Res*. 2022;15(1):1–12.

[15] ICH E9 Working Group, International Council for Harmonisation of Technical Requirements for Pharmaceuticals for Human Use . ICH E9 (R1) Addendum on Estimands and Sensitivity Analysis in Clinical Trials to the Guideline on Statistical Principles for Clinical Trials. Amsterdam, the Netherlands: European Medicines Agency; 2020. https://www.ema.europa.eu/en/documents/scientific-guideline/ich-e9-r1-addendum-estimands-sensitivity-analysis-clinical-trials-guideline-statistical-principles_en.pdf. Accessed September 1, 2022.

[16] Schulz KF , Altman DG, Moher D. CONSORT 2010 statement: updated guidelines for reporting parallel group randomised trials. *BMJ*. 2010;340:c332. 20332509 10.1136/bmj.c332 PMC2844940

[17] Chan AW , Tetzlaff JM, Altman DG, et al. SPIRIT 2013 statement: defining standard protocol items for clinical trials. *Ann Intern Med*. 2013;158(3):200–207. 23295957 10.7326/0003-4819-158-3-201302050-00583 PMC5114123

[18] von Elm E , Altman DG, Egger M, et al. The Strengthening the Reporting of Observational Studies in Epidemiology (STROBE) statement: guidelines for reporting observational studies. *Lancet*. 2007;370(9596):1453–1457. 18064739 10.1016/S0140-6736(07)61602-X

[19] Page MJ , McKenzie JE, Bossuyt PM, et al. The PRISMA 2020 statement: an updated guideline for reporting systematic reviews. *BMJ*. 2021;372:n71. 33782057 10.1136/bmj.n71 PMC8005924

[20] Altman DG . Better reporting of randomised controlled trials: the CONSORT statement. *BMJ*. 1996;313(7057):570–571. 8806240 10.1136/bmj.313.7057.570 PMC2352018

[21] Clark TP , Kahan BC, Phillips A, et al. Estimands: bringing clarity and focus to research questions in clinical trials. *BMJ Open*. 2022;12(1):e052953. 10.1136/bmjopen-2021-052953 PMC8724703 34980616

[22] Fiero MH , Pe M, Weinstock C, et al. Demystifying the estimand framework: a case study using patient-reported outcomes in oncology. *Lancet Oncol*. 2020;21(10):e488–e494. 33002444 10.1016/S1470-2045(20)30319-3

[23] Lawrance R , Degtyarev E, Griffiths P, et al. What is an estimand & how does it relate to quantifying the effect of treatment on patient-reported quality of life outcomes in clinical trials? *J Patient Rep Outcomes*. 2020;4(1):68. 32833083 10.1186/s41687-020-00218-5 PMC7445213

[24] Rufibach K . Treatment effect quantification for time-to-event endpoints—estimands, analysis strategies, and beyond. *Pharm Stat*. 2019;18(2):145–165. 30478869 10.1002/pst.1917

[25] Sun S , Weber HJ, Butler E, et al. Estimands in hematologic oncology trials. *Pharm Stat*. 2021;20(4):793–805. 33686762 10.1002/pst.2108

[26] Frangakis CE , Rubin DB. Principal stratification in causal inference. *Biometrics*. 2002;58(1):21–29. 11890317 10.1111/j.0006-341x.2002.00021.x PMC4137767

[27] Kahan BC , Li F, Copas AJ, et al. Estimands in cluster-randomized trials: choosing analyses that answer the right question. *Int J Epidemiol*. 2023;52(1):107–118. 35834775 10.1093/ije/dyac131 PMC9908044

[28] Kahan BC , White IR, Eldridge S, et al. Independence estimators for re-randomisation trials in multi-episode settings: a simulation study. *BMC Med Res Methodol*. 2021;21(1):235. 34717559 10.1186/s12874-021-01433-4 PMC8557515

[29] Kahan BC , White IR, Hooper R, et al. Re-randomisation trials in multi-episode settings: estimands and independence estimators. *Stat Methods Med Res*. 2022;31(7):1342–1354. 35422159 10.1177/09622802221094140 PMC9251752

[30] Li H , Wang C, Chen W-C, et al. Estimands in observational studies: some considerations beyond ICH E9 (R1). *Pharm Stat*. 2022;21(5):835–844. 35128808 10.1002/pst.2196

[31] Cro S , Cornelius VR, Pink AE, et al. Anakinra for palmoplantar pustulosis: results from a randomized, double-blind, multicentre, two-staged, adaptive placebo-controlled trial (APRICOT). *Br J Dermatol*. 2021;186(2):245–256. 34411292 10.1111/bjd.20653 PMC9255857

[32] International Council for Harmonisation of Technical Requirements for Pharmaceuticals for Human Use . ICH guideline implementation. https://www.ich.org/page/ich-guideline-implementation. Accessed September 1, 2022.

[33] Homer V , Yap C, Bond S, et al. Early phase clinical trials extension to guidelines for the content of statistical analysis plans. *BMJ*. 2022;376:e068177. 35131744 10.1136/bmj-2021-068177 PMC8819597

[34] Wilding JPH , Batterham RL, Calanna S, et al. Once-weekly semaglutide in adults with overweight or obesity. *N Engl J Med*. 2021;384(11):989–1002. 33567185 10.1056/NEJMoa2032183

[35] Frias JP , Bonora E, Nevarez Ruiz L, et al. Efficacy and safety of dulaglutide 3.0 mg and 4.5 mg versus dulaglutide 1.5 mg in metformin-treated patients with type 2 diabetes in a randomized controlled trial (AWARD-11). *Diabetes Care*. 2021;44(3):765–773. 33397768 10.2337/dc20-1473 PMC7896253

[36] Aroda VR , Rosenstock J, Terauchi Y, et al. PIONEER 1: randomized clinical trial of the efficacy and safety of oral semaglutide monotherapy in comparison with placebo in patients with type 2 diabetes. *Diabetes Care*. 2019;42(9):1724–1732. 31186300 10.2337/dc19-0749

[37] Goodwin GM , Aaronson ST, Alvarez O, et al. Single-dose psilocybin for a treatment-resistant episode of major depression. *N Engl J Med.* 2022;387(18):1637–1648. 36322843 10.1056/NEJMoa2206443

[38] Li F , Li F. Propensity score weighting for causal inference with multiple treatments. *Ann Appl Stat*. 2019;13(4):2389–2415.

[39] Li F , Morgan KL, Zaslavsky AM. Balancing covariates via propensity score weighting. *J Am Stat Assoc*. 2018;113(521):390–400.

[40] Li F , Thomas LE, Li F. Addressing extreme propensity scores via the overlap weights. *Am J Epidemiol*. 2019;188(1):250–257. 30189042 10.1093/aje/kwy201

[41] Reilly MC , Zbrozek AS, Dukes EM. The validity and reproducibility of a work productivity and activity impairment instrument. *Pharmacoeconomics*. 1993;4(5):353–365. 10146874 10.2165/00019053-199304050-00006

