# Supplementary material for: Eliminating Ambiguous Treatment Effects Using Estimands
Source: Am J Epidemiol. 2023 Feb 14;192(6):987–94. doi: 10.1093/aje/kwad036 (PMC10236519; doi:10.1093/aje/kwad036)
Supplement: Web_Material_kwad036 [file web_material_kwad036.pdf]

WEB MATERIAL

Eliminating Ambiguous Treatment Effects Using Estimands

Brennan C. Kahan*, Suzie Cro, Fan Li, and Michael O. Harhay

* (b.kahan@ucl.ac.uk)

Table of Contents

Item	Page No.
**Web Appendix 1**. Trial of baricitinib for atopic dermatitis	2
**Web Table 1**. Example of reporting estimands for the trial of baricitinib for atopic dermatitis	2
**Web Appendix 2**. Trial of anakinra for palmoplantar pustulosis	3
**Web Table 2**. Example of reporting estimands for the trial of anakinra for palmoplantar pustulosis (primary estimand)	3
**Web Table 3**. Example of reporting estimands for the trial of anakinra for palmoplantar pustulosis (supplementary estimand)	4

WEB APPENDIX 1

Trial of Baricitinib for Atopic Dermatitis

The primary estimand aimed to address the effect of baricitinib vs. placebo in the hypothetical setting where participants did not discontinue treatment and could not receive rescue therapy. Full details are provided in Web Table 1.

Web Table 1. Example of reporting estimands for the trial of baricitinib for atopic dermatitis

Estimand Aspect	Definition
Population	Adults with atopic dermatitis (meeting the trial eligibility criteria)
Treatment conditions	Baricitinib 4-mg or 2-mg vs. placebo daily plus topical corticosteroids, without discontinuation and without rescue therapy
Endpoint	WPAI-AD change from baseline score at 16 weeks
Summary measure	Difference in means
Handing of intercurrent events	
Incorrect treatment received	Treatment policy strategy
Treatment discontinuation	Hypothetical strategy, pertaining to the setting where participants are kept on treatment regardless of side effects or other adverse effects
Use of rescue therapy	Hypothetical strategy, pertaining to the setting where rescue therapy is not provided even if medically indicated

WEB APPENDIX 2

Trial of Anakinra for Palmoplantar Pustulosis

The primary estimand aimed to address the effect of anakinra vs. placebo regardless of treatment discontinuation or receipt of additional therapies. Full details are provided in Web Table 2. A supplementary estimand addressed the effect of anakinra vs. placebo in the subset of participants who would receive at least 50% of daily injections under both treatment conditions, regardless of receipt of additional therapies. Full details are provided in Web Table 3 (see next page).

Web Table 2. Example of reporting estimands for the trial of anakinra for palmoplantar pustulosis (primary estimand)

Estimand Aspect	Definition
Population	Patients with confirmed diagnosis of PPP meeting the trial eligibility criteria
Treatment conditions	8 weeks of treatment with anakinra vs. placebo, regardless of treatment discontinuation or receipt of additional therapies
Endpoint	Change from baseline in PPPASI at week 8
Summary measure	Mean difference
Handing of intercurrent events	
Study treatment discontinuation	Treatment policy
Use of rescue medication	Treatment policy
Use of prohibited medication	Treatment policy
Use of other topical medication	Treatment policy

PPP, palmoplantar pustulosis; PPPASI, palmoplantar pustulosis area and severity score.

Web Table 3. Example of reporting estimands for the trial of anakinra for palmoplantar pustulosis (supplementary estimand)

Estimand Aspect	Definition
Population	Patients with confirmed diagnosis of PPP meeting the trial eligibility criteria who would comply with treatment and receive at least 50% of daily injections
Treatment conditions	8 weeks of treatment with anakinra vs. placebo, regardless of treatment discontinuation or receipt of additional therapies
Endpoint	Change from baseline in PPPASI at week 8
Summary measure	Mean difference
Handing of intercurrent events	
Study treatment discontinuation (received <50% of daily injections)	Principal stratum of participants who would receive at least 50% of daily injections under either treatment condition
Study treatment discontinuation (received ≥ 50% of daily injections)	Treatment policy
Use of rescue medication	Treatment policy
Use of prohibited medication	Treatment policy
Use of other topical medication	Treatment policy

PPP, palmoplantar pustulosis; PPPASI, palmoplantar pustulosis area and severity score.
